# A Novel Six Metastasis-Related Prognostic Gene Signature for Patients With Osteosarcoma

**DOI:** 10.3389/fcell.2021.699212

**Published:** 2021-07-23

**Authors:** Di Zheng, Kezhou Xia, Ling Yu, Changtian Gong, Yubo Shi, Wei Li, Yonglong Qiu, Jian Yang, Weichun Guo

**Affiliations:** Department of Orthopedics, Renmin Hospital of Wuhan University, Wuhan, China

**Keywords:** epithelial to mesenchymal transition, FHIT, metastasis, osteosarcoma, prognostic model

## Abstract

Osteosarcoma is the most common malignant bone tumor, and although there has been significant progress in its management, metastases often herald incurable disease. Here we defined genes differentially expressed between primary and metastatic osteosarcoma as metastasis-related genes (MRGs) and used them to construct a novel six-MRG prognostic signature for overall survival of patients with osteosarcoma. Validation in internal and external datasets confirmed satisfactory accuracy and generalizability of the prognostic model, and a nomogram based on the signature and clinical variables was constructed to aid clinical decision-making. Of the six MRGs, *FHIT* is a well-documented tumor suppressor gene that is poorly defined in osteosarcoma. Consistent with tumor suppressor function, FHIT was downregulated in osteosarcoma cells and human osteosarcoma samples. FHIT overexpression inhibited osteosarcoma proliferation, migration, and invasion both *in vitro* and *in vivo*. Mechanistically, FHIT overexpression upregulate the epithelial marker E-cadherin while repressing the mesenchymal markers N-cadherin and vimentin. Our six-MRG signature represents a novel and clinically useful prognostic biomarker for patients with osteosarcoma, and FHIT might represent a therapeutic target by reversing epithelial to mesenchymal transition.

## Introduction

Osteosarcoma is the most common primary malignant bone tumor. It mainly occurs in children and adolescents with an incidence of approximately one to four cases per million each year (Kansara et al., 2014; Gianferante et al., 2017; Whelan and Davis, 2018). Osteosarcoma usually arises from mesenchymal stem cells in the metaphyses of long bones such as the distal femur, proximal tibia, and proximal humerus (Ritter and Bielack, 2010; Deng et al., 2019). Osteosarcoma is highly aggressive and consequently incurs significant morbidity and mortality. There has been significant progress in the diagnosis and treatment of osteosarcoma over the last thirty years, which has positively impacted survival. The current standard of care for osteosarcoma is neoadjuvant chemotherapy with doxorubicin, cisplatin, and methotrexate combined with surgical resection (Prudowsky and Yustein, 2020; Marchandet et al., 2021), which, for early stage patients, has increased 5-year survival rates from 10 to 60–70% (Whelan and Davis, 2018). However, patients with relapsed and metastatic osteosarcoma—the main cause of death in osteosarcoma patients—still represent a management challenge, and their 5-year survival has remained unchanged at ∼15–30% (Kansara et al., 2014; Lin et al., 2017). Unfortunately, approximately 10–20% of osteosarcoma patients have detectable metastases at diagnosis, with the lung is the most common site of metastasis (Harrison et al., 2018). Determining the natural history and prognosis of osteosarcoma patients remains difficult due to a lack of reliable biomarkers, and novel strategies and reliable prognostic markers for osteosarcoma are urgently required. Determining the molecular mechanisms underlying osteosarcoma metastasis and identifying novel therapeutic targets or biomarkers would provide novel strategies for the treatment and prognostication of patients with osteosarcoma.

Over the last decade, high-throughput sequencing has revolutionized cancer genetics and provided comprehensive molecular portraits of osteosarcoma evolution (Gianferante et al., 2017; Rickel et al., 2017; Sayles et al., 2019; Zhao et al., 2020). Through the analysis of high-throughput sequencing data, a number of genetic and epigenetic abnormalities have been discovered and experimentally confirmed to be associated with osteosarcoma progression and may therefore be good prognostic biomarkers (Wu et al., 2020; Zhou et al., 2020). For example, in their analysis of TCGA data, Wu et al. (2020) reported that high expression of *FGD1* was associated with poor prognosis in osteosarcoma patients and confirmed higher protein expression of FGD1 in osteosarcoma tissues than in adjacent normal tissues. Functionally, aberrant FGD1 expression might promote tumor cell proliferation and invasion by activating PI3K/AKT signaling. Liu et al. (2019) constructed a two-gene signature using seed genes (coefficient of variation larger than 20%) identified in gene expression data from the Gene Expression Omnibus (GEO) database and confirmed it as an independent prognostic marker in osteosarcoma. However, the full repertoire of genes driving osteosarcoma progression is unknown.

Motivated by these previous efforts and a need for a prognostic biomarker, here we analyzed the expression profiles of osteosarcoma samples obtained from the GEO database and identified genes that were differentially expressed (DEGs) between primary and metastatic osteosarcoma samples. We hypothesized that these DEGs were associated with metastasis and so defined them as metastasis-related genes (MRGs), which we subjected to functional analysis. A prognostic model based on six MRGs was constructed and validated in the internal TCGA osteosarcoma cohort and external GSE39055 cohort. Of the six MRGs, *FHIT* was a potent tumor suppressor that was downregulated in osteosarcoma cell lines and tissue samples. The effects of FHIT on osteosarcoma cell proliferation, migration, and invasion were examined *in vitro* and *in vivo* and the mechanism of action of FHIT in osteosarcoma metastasis further explored.

## Materials and Methods

### Data Collection

The gene matrices of 84 osteosarcoma samples and corresponding clinical information were downloaded from the TARGET database.^1^ Samples without follow-up were excluded to produce a final dataset of 81 osteosarcoma samples (TCGA cohort) for subsequent analysis. The GSE39055 cohort containing 37 osteosarcoma samples with clinical follow-up was downloaded from the National Center for Biotechnology Information (NCBI) GEO repository.^2^ The clinicopathological characteristics of the TCGA and GSE39055 cohorts are shown in Table 1. GSE14359 and GSE32981 datasets containing primary and metastatic osteosarcoma were download from the GEO database, with GSE14359 containing 10 primary OS samples and 8 metastatic OS samples and GSE32981 containing 12 primary OS samples and 11 metastatic OS samples.

**TABLE 1:** Clinicopathological characteristics of osteosarcoma patients used in this study.

Clinical characteristics	TCGA cohort	GSE39055 dataset
**No. of patients**	81	34
**Age (median, range)**	14 (4–33)	11 (4–71)
**Gender (%)**		
Female	37 (45.7)	17 (45.9)
Male	44 (54.3)	20 (54.1)
**Ethnicity (%)**		
Asian	7 (8.6)	NA
White	49 (60.5)	NA
Black or African American	6 (7.4)	NA
Unknown	19 (23.5)	NA
**Site (%)**		
Lower limb	75 (92.6)	NA
Upper limb	6 (7.4)	NA
**Necrosis (%)**		
≤ 50%	NA	16 (43.2)
> 50%	NA	21 (56.8)
**Recurrence (%)**		
No	NA	19 (51.4)
Yes	NA	18 (48.6)
**Survival status**		
OS days (median)	1468	1019
Censored (%)	26 (32.1)	10 (27.0)

### Differential Analysis

Differentially expressed genes (DEGs) between primary and metastatic osteosarcoma samples were identified using R software and *limma* package. DEGs were identified with a cutoff of | log2 (fold change)| > 0.5 and *P* < 0.05. Shared DEGs in the GSE14359 dataset and GSE32981 dataset were defined as MRGs.

### Functional Analysis

We conducted GO (gene ontology) and KEGG (Kyoto Encyclopedia of Genes and Genomes) pathway enrichment analysis on 160 identified MRGs and DEGs between high- and low-risk groups using DAVID (Database for Annotation, Visualization, and Integrated Discovery). A *P*-value < 0.05 was regarded as significant enrichment. Gene set enrichment analysis (GSEA) software was used to interrogate the gene expression matrices of the TCGA and GSE39055 cohorts.

### PPI Network Construction

MRGs were used to construct a protein-protein interaction (PPI) network using the STRING database.^3^ Cytoscape software was used to reconstruct and visualize the network. Hub genes were identified using the *cytohubba* plugin. The number of adjacent nodes of the MRGs were visualized in R.

### Construction and Validation of the MRG-Based Prognostic Signature

**FIGURE 1:**
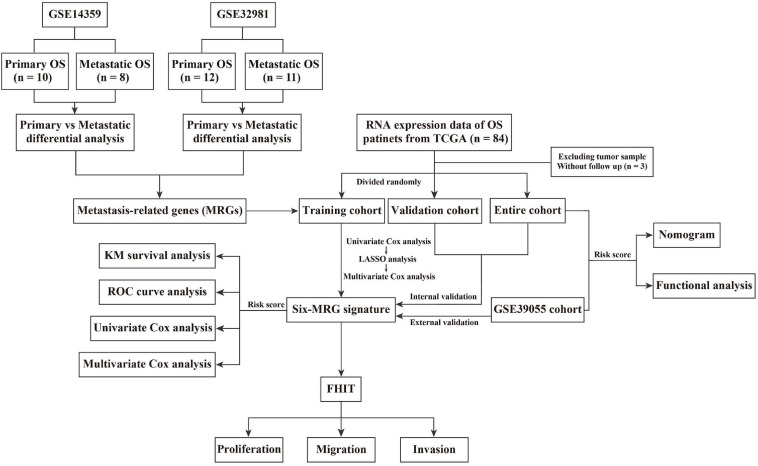
Flowchart of the process used to derive the six-MRG signature and the survival analysis.

Details of the construction of the MRG-based prognostic signature are shown in Figure 1. Briefly, the TCGA cohort was randomly divided into a training cohort and a validation cohort in an approximately 1:1 ratio. Univariate Cox proportional hazard regression analysis was performed to detected prognostic genes. Then, Lasso (least absolute shrinkage and selector operator) regression analysis was carried out to identify the most powerful prognostic genes using the *glmnet* package in R, followed by stepwise multivariate Cox regression to construct the MRG-based prognostic signature. The risk score was calculated using the following formula: Risk score = $\sum_{\text{i}}^{\text{n}}{\text{Exp}}_{\text{i}}\,{\text{Coe}}_{\text{i}}$ (Exp = expression level; Coe = regression coefficient). Patients in the training cohort, validation cohort, entire cohort, and external GSE39055 cohort were divided into low- and high-risk groups based on the median risk score value in the training cohort. Kaplan-Meier (KM) survival analysis was performed to assess differences in overall survival between the low- and high-risk groups using the *survival* package in R. Time-dependent receiver operating characteristic (ROC) curve analysis was carried out to compute the sensitivity and specificity of the six-MRG signature in predicting the survival of osteosarcoma patients using the *survivalROC* package in R.

### Tissue Collection

Twenty-four frozen tissue samples, including 12 osteosarcoma tissues and 12 matched adjacent normal tissues, were collected from patients with a histopathological diagnosis of osteosarcoma undergoing surgery in Renmin Hospital of Wuhan University between June 2018 and July 2020. These samples were harvested immediately after surgical resection and stored in liquid nitrogen until RNA or protein extraction. All patients signed informed consent, and the research the Research Ethics Committee of Renmin Hospital of Wuhan University approved the study protocol.

### Osteosarcoma Cell Lines and Lentivirus Infection

The human osteoblast cell line hFOB1.19 and human osteosarcoma cell lines 143B, MG63, U2OS, and HOS were purchased from the Cell Bank of Type Culture Collection (CBTCC, Chinese Academy of Sciences, Shanghai, China) and Cell Bank of Wuhan University (Wuhan, China). hFOB1.19 cells were cultured in DMEM/F-12 complete medium. U2OS cells were cultured in McCoy’s 5a complete medium, while MG63 and 143B cells were cultured in α-MEM complete medium. All complete media were supplemented with 10% FBS (Gibco, Thermo Fisher Scientific, Waltham, MA, United States) and 1% antibiotics (100 units/mL penicillin and 100 units/mL streptomycin). All cells were maintained in a humidified incubator at 37°C and 5% CO_2_ atmosphere. To construct stable osteosarcoma cell lines overexpressing *FHIT*, lentivirus containing pLVX-FHIT-Puro and pLVX-Puro (vector) were purchased from Genechem (GeneChem, Shanghai, China) and were used to infect 143B and MG63 cells according to the manufacturer’s protocol. Stably transfected osteosarcoma cells were selected using puromycin (1.5 μg/mL) and confirmed by western blotting and qRT-PCR.

### CCK-8 and Colony Formation Assays

CCK-8 (Cell Counting Kit-8) and colony formation assays were carried out to assess osteosarcoma cell proliferation. For the CCK-8 assay, *FHIT*-overexpressing and control 143B and MG63 cells were seeded in 96-well plates in triplicate at a density of 10^4^ cells/well. The CCK-8 reagent (Dojindo Laboratories Co., Ltd., Kumamoto, Japan) was added to wells at the indicated timepoints. The plate was incubated for 1 h at 37°C before reading the optical density (OD) at 450 nm. For the colony formation assay, stably transfected 143B and MG63 cells were harvested and seeded into six-well plates at a density of 5 × 10^2^ cells/well. Cells were cultured for 2 weeks, with the medium replaced every 3 days. After 2 weeks, colonies were fixed and stained with 1% crystal violet. The plates were photographed, and the number of colonies in every well was counted.

### Wound Healing and Transwell Invasion Assays

Wound healing and transwell invasion assays were performed to evaluate the migration and invasion of osteosarcoma cells. For the wound healing assay, transfected osteosarcoma cells were seeded in six-well plates. When the cell density reached approximately 90%, cells were scratched with a 200 μL sterile pipette tip to create a gap and cultured in serum-free medium for 48 h. The gaps were observed and photographed at 0 and 48 h by inverted microscopy (Olympus, Tokyo, Japan). Matrigel chambers (Corning Costar, Corning, NY, United States) were used for the transwell invasion assay. Briefly, transfected osteosarcoma cells were digested and re-suspended in serum-free medium at a density of 10^6^ cells/mL. Hundred microliter of cell suspension was added into the upper chamber, and 600 μL of medium (20% FBS) was added into the sub-chamber. 48 h later, cells in the bottom chamber were fixed and stained with 1% crystal violet before being photographed by inverted microscopy. The number of migrating cells was analyzed by ImagePro Plus 5.0 (Media Cybernetics, Rockville, MD, United States).

### RNA Isolation and qRT-PCR

Total RNA from osteosarcoma samples and cells was isolated using TRIzol reagent (Invitrogen, Carlsbad, CA, United States) according to the manufacturer’s protocol. One microgram total RNA was reverse transcribed into cDNA using the RevertAid First Strand cDNA Synthesis Kit (Thermo Fisher Scientific). Then, qRT-PCR was conducted using the SYBR Green Mix (Vazyme, China). Relative gene expression was calculated by the 2^–ΔΔ*Ct*^ method, and *GAPDH* expression served as an internal control. The primer sequences for qRT-PCR are listed as follow: GAPDH, forward, 5′-CTGAGTACGTCGTGGAGTCC-3′ and reverse, 5′-GTCTTCTGGGTGGCAGTGAT-3′; FHIT, forward, 5′-GCCAACATCTCATCA-AGCCC-3′ and reverse, 5′-AATCG GCCACTTCATCAGGA-3′; E-cadherin, forward, 5′-ACGCAT- TGCCACATACACTC-3′ and reverse, 5′-GGTGTTCACATC ATCGTCCG-3′; N-cadherin, forward, 5′-CCATCATTGCCATC CTGCTC-3′ and reverse, 5′-ATCAGGCTCCACAGTGTCAG-3′; vimentin, forward, 5′-GAGTCCACTGAGTACCGGAG-3′ and reverse, 5′-ACGAGCCATTTCCT-CCTTCA-3′

### Western Blotting

Briefly, proteins were extracted from osteosarcoma samples and cells using RIPA lysis buffer (Cell Signaling Technology, Danvers, MA, United States), and the protein concentration was determined using the BCA protein assay. Proteins were separated by 10% SDS-PAGE gel electrophoresis and then transferred onto PVDF membranes. After blocking with 5% skimmed milk, membranes were incubated with specific antibodies at 4°C overnight. The next day, membranes were washed twice with TBST and incubated with horseradish peroxidase-linked secondary antibodies (1:10,000) at room temperature for 1 h. Blots were visualized using the ECL Kit (Servicebio, Wuhan, China) and analyzed with ImageJ software. Antibodies targeting GAPDH, Ki67, E-cadherin, N-cadherin, and vimentin were purchased from Cell Signaling Technology, and antibodies targeting FHIT were purchased from Abcam (Cambridge, United Kingdom).

### Xenograft Tumor Model

Four-week-old male BALB/c nude mice were purchased from the Beijing HFK Experiment Animal Center (Beijing, China) and housed in the Animal Center of Wuhan University Renmin Hospital. Nude mice were injected subcutaneously with 5 × 10^6^ stably transfected 143B cells or control cells. Tumor volumes were monitored using calipers every 7 days, and tumor volume was calculated as follows: Volume (mm^3^) = (length × width^2^)/2. Five weeks after cell injection, all mice were sacrificed and the xenografts weighed and harvested. All animal experiments were approved by the Animal Care and Use Committee of Wuhan University Renmin Hospital.

### Immunohistochemistry Assay

Subcutaneous tumor tissues were fixed with 4% paraformaldehyde and embedded in paraffin. Then, 4 μm-thick tissue sections were stained with primary antibodies against target antigens (Ki67, E-cadherin, N-cadherin, and vimentin, all from Cell Signaling Technology) and a horseradish peroxidase-conjugated IgG (Servicebio). Proteins were visualized with DAB (3,3-diaminobenzidine), and the sections were mounted using transparent xylene. Positive brown staining was observed and photographed by light microscopy.

### Statistical Analysis

All statistical analyses were conducted using R software (3.6.1) and GraphPad Prism version 8.0 software (GraphPad Software, La Jolla, CA, United States). All experiments were performed at least three times independently, and results are presented as means ± standard deviation (SD). Student’s *t*-test was used to analyze differences between two groups and one-way ANOVA was used to compare more than two groups. A *P*-value < 0.05 was regarded as statistically significant.

## Results

### Identification of Metastasis-Related Genes and Functional Enrichment Analysis

**FIGURE 2:**
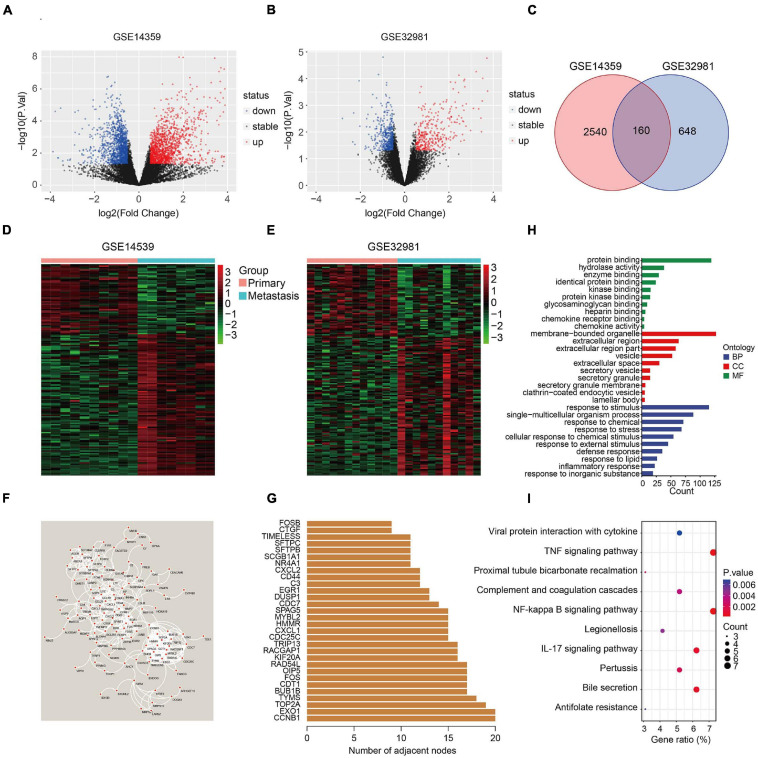
Identification of metastasis-related genes and functional enrichment analysis. **(A,B)** Volcano plot showing differentially expressed genes between primary and metastatic osteosarcoma. **(C)** Venn diagram of metastasis-related genes in osteosarcoma. **(D,E)** Heatmaps of metastasis-related genes in the GSE14539 and GSE32981 datasets. **(F)** Construction of a protein-protein interaction network using the metastasis-related genes. **(G)** The number of adjacent nodes of the metastasis-related genes. **(H)** GO analysis of the metastasis-related genes. **(I)** KEGG pathway enrichment analysis of the metastasis-related genes.

The flowchart of the study is presented in Figure 1. First, two osteosarcoma datasets were identified in the GEO database, both of which contained primary and metastatic osteosarcoma samples. There were 1448 upregulated and 1252 downregulated DEGs in GSE14359 and 348 upregulated and 460 downregulated DEGs in GSE32981 (Figures 2A,B). Of these, 160 DEGs were common to both datasets, so we defined these shared DEGs as MRGs (Figure 2C).

The expression of the 160 MRGs in the two GEO datasets is shown in Figures 2D,E and a protein-protein interaction (PPI) network for the 160 MRGs in Figure 2F. Hub gene analysis indicated that *CCNB1* and *EXO1* were the top two ranked genes in the PPI network (Figure 2G). GO analysis showed that response to stimulus, response to stress, response to external stimulus, defense response, and inflammatory response were significantly enriched processes in the 160 MRGs. In the cellular component (CC), MRGs were particularly enriched for membrane-bound organelles, extracellular regions, vesicles, extracellular space, and secretory vesicles. In terms of molecular function (MF), protein binding, hydrolase activity, and enzyme binding were the three most enriched terms (Figure 2H). KEGG pathway enrichment analysis revealed that MRGs primarily participated in the TNF and NF-kappa B signaling pathways (Figure 2I), two signaling pathways known drive metastasis in other cancers.

### Construction of a Six-MRG Prognostic Model in the TCGA Osteosarcoma Cohort

**FIGURE 3:**
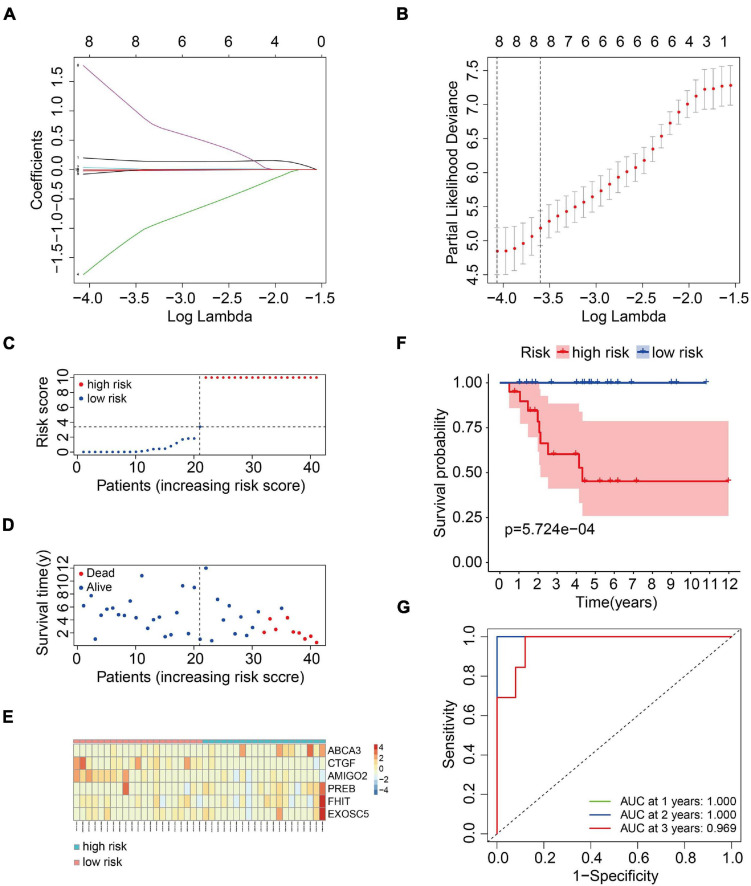
Construction of a six-MRG prognostic model in the training cohort. **(A,B)** Lasso regression and stepwise multivariate Cox regression were used to construct the six-MRG signature. **(C)** The risk score distribution of patients in the training cohort. **(D)** The survival status of patients in the training cohort. **(E)** mRNA expression profiles of the six MRGs in the training cohort. **(F)** Kaplan-Meier survival analysis of patients in the training cohort. **(G)** ROC curve analysis of the six-MRG signature in the training cohort.

To construct a prognostic model using the MRGs, the TCGA osteosarcoma cohort was randomly divided into a training cohort and a validation cohort at a ratio of approximately 1:1. In the training cohort, univariate Cox and Lasso regression analyses were conducted to determine the most powerful prognostic markers of the 160 MRGs (Figures 3A,B). Then, stepwise multivariate Cox regression analysis was performed to ultimately construct a six-MRG prognostic signature for osteosarcoma patients. The six MRGs were *ABCA3*, *CTGF*, *AMIGO2*, *PREB*, *FHIT*, and *EXOSC5*. The regression coefficients of the six MRGs are shown in Table 2, and the expression of the six MRGs in the training cohort are shown in Figure 3E.

**TABLE 2:** Details of the six MRGs in the prognostic model.

Gene name	Coefficient	HR	HR.95L	HR.95H	*P*
*ABCA3*	0.566603	1.762270	0.761259	4.079553	0.185829
*CTGF*	–0.057000	0.944597	0.854470	1.044231	0.265271
*AMIGO2*	–5.564460	0.003832	1.33E-05	1.102538	0.054083
*PREB*	0.175290	1.191592	0.970820	1.462571	0.093603
*FHIT*	6.429405	619.8050	0.833831	460714.9	0.056639
*EXOSC5*	–0.409400	0.664048	0.437241	1.008506	0.054828

Next, a risk score was calculated for each patient in the training cohort as follows: Risk score = (*ABCA3* × 0.566 + *CTGF* × –0.057 + *AMIGO2* × –5.564 + *PREB* × 0.175 + *FHIT* × 6.429 + *EXOSC5* × –0.409). Taking the median value of the risk score as the cutpoint, patients in the training cohort were separated into low- and high-risk groups (Figure 3C). As shown in Figures 3D,E, patients in the high-risk group tended to have a shorter survival time and higher risk of death. Kaplan-Meier survival curve analysis showed that patients in high-risk group had significantly poorer survival than patients in the low-risk group (Figure 3F). To evaluate the predictive power of the six-MRG signature, time-dependent ROC curve analysis was carried out. As shown in Figure 3G, the area under the curve (AUC) was 1.00 at 1 year, 1.00 at 2 years, and 0.969 as 3 years, suggesting that the six-MRG signature effectively determined the prognosis of osteosarcoma patients.

### Validation of the Six-MRG Prognostic Model in Internal and External Cohorts

**FIGURE 4:**
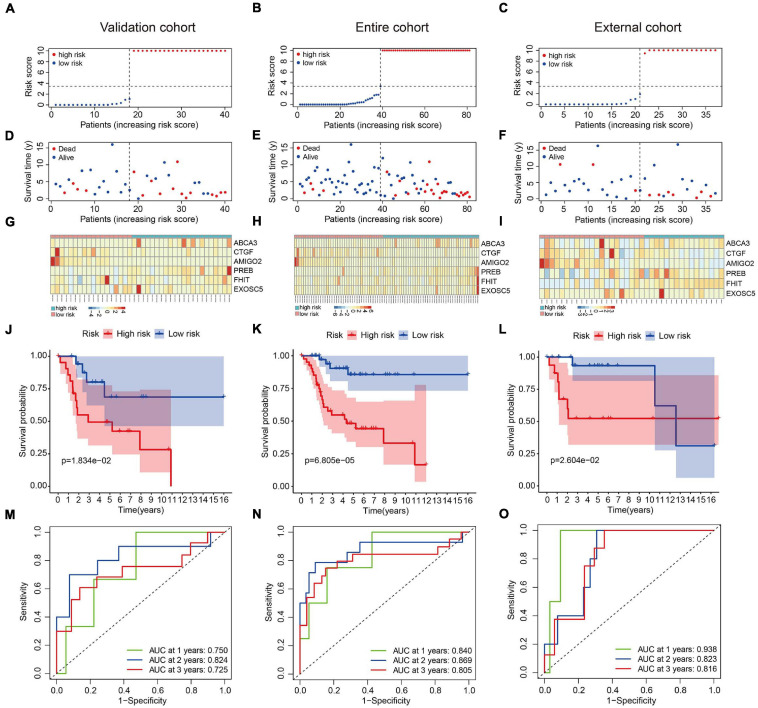
Validation of the six-MRG prognostic model in the internal and external cohorts. **(A–C)** The risk score distribution of patients in the validation cohort, entire cohort, and external cohort. **(D–F)** The survival status of osteosarcoma patients in the three cohorts. **(G–I)** The mRNA expression profiles of the six MRGs in the three cohorts. **(J–L)** Kaplan-Meier survival analysis of patients in the three cohorts. **(M–O)** ROC curve analysis of the six-MRG signature in the three cohorts.

We used an internal validation cohort, the entire cohort, and an external cohort to assess the reliability and stability of the six-MRG signature for prognostication. Using the same formula as above, the risk scores were calculated for patients in these cohorts. Then, patients were stratified into high- and low-risk groups according to the previously established median value in the training cohort. The risk score distribution of patients in the three cohorts are shown in Figures 4A–C, survival in Figures 4D–F, and the expression levels of the six MRGs in Figures 4G–I. Kaplan-Meier survival curve analysis confirmed that patients in the high-risk group had consistently and significantly poorer overall survival than patients in the low-risk group in all three cohorts (Figures 4J–L). The predictive power of the six-MRG signature was also evaluated using ROC curve analysis in the internal and external cohorts. In the validation cohort, the 1-, 2-, and 3-years overall survival AUCs of the six-MRG signature were 0.750, 0.824, and 0.725, respectively (Figure 4M). In the TCGA entire cohort, the 1-, 2-, and 3-years overall survival AUCs were 0.840, 0.869, and 0.805, respectively (Figure 4N). Furthermore, in the GSE39055 external cohort, the 1-, 2-, and 3-years overall survival AUCs were 0.938, 0.823, and 0.816, respectively (Figure 4O). Taken together, these validation results demonstrate satisfactory accuracy and generalizability of the prognostic model based on the six-MRG signature in osteosarcoma patients.

### The Six-MRG Signature Is Independent of Clinical Factors and Associated With Poor Prognosis and Adverse Clinical Features

**FIGURE 5:**
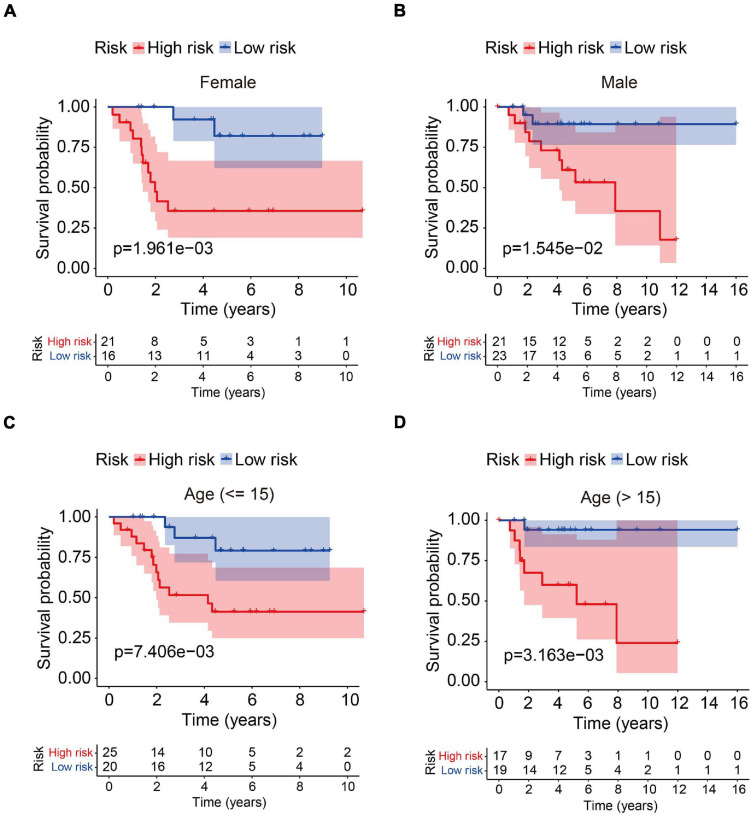
Analysis of the six-MRG signature in clinicopathological subtypes of osteosarcoma. **(A,B)** Kaplan-Meier survival curve analysis of high- and low-risk males and females. **(C,D)** Kaplan-Meier survival curve analysis of high- and low-risk patients aged ≤ 15 or age > 15 years.

In order to assess whether the six-MRG signature was independent of other clinical factors including gender, age, and tumor site, univariate and multivariate Cox regression analyses were performed in the TCGA osteosarcoma cohort (Table 3). The risk score was an independent prognostic marker in patients with osteosarcoma. We further examined the predictive value of the six-MRG signature in predicting outcomes in patients with different clinical features. Patients in the TCGA osteosarcoma cohort were divided into various clinical sub-groups including female and male and aged ≤ 15 and > 15 years before survival analysis according to high- and low-risk groups. Kaplan-Meier survival curve analysis showed that patients in the high-risk group had poorer overall survival than those in low-risk group in all clinical sub-groups (Figure 5).

**TABLE 3:** Univariable and multivariable analysis of the six-MRG signature and clinical factors in the TCGA osteosarcoma cohort.

Variables	Univariable analysis				Multivariable analysis			
	
	HR	95% CI of HR		*P*	HR	95% CI of HR		*P*
							
		Lower	Upper			Lower	Upper	
Gender (Female vs. Male)	0.546166519	0.24754378	1.205030749	0.134124815	0.539832412	0.218377899	1.334471275	0.181842468
Age (≤ 15 vs. > 15)	0.985348892	0.900215058	1.078533879	0.748865907	1.022160398	0.92253064	1.132549785	0.675290481
Site (lower limb vs. upper limb)	1.245115163	0.292335236	5.303198453	0.766834464	1.244072038	0.288740181	5.360235046	0.769482752
Risk score	1.000000003	1.000000001	1.000000005	0.001959531	1.000000003	1.000000001	1.000000005	0.003580904

### Construction and Validation of a Nomogram in the TCGA Osteosarcoma Cohort

**FIGURE 6:**
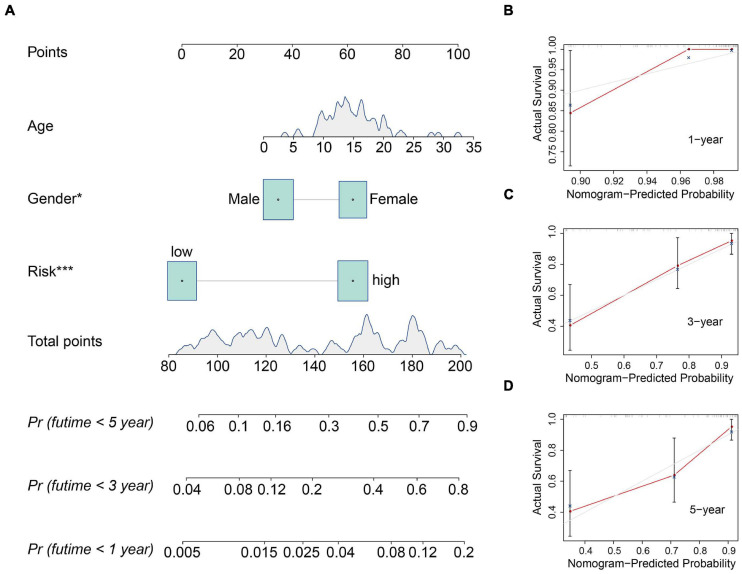
Construction and validation of a nomogram in the TCGA osteosarcoma cohort. **(A)** The nomogram consists of age, gender, and risk score based on the six-MRG signature. **(B–D)** The calibration curve for internal validation of the nomogram for estimating the survival of osteosarcoma patients at 1, 3, and 5 years.

We also construct a nomogram based on the TCGA osteosarcoma cohort to facilitate clinical decision-making. Clinicopathological characteristics including age, gender, tumor site, and risk score were all included in the nomogram (Figure 6A). As shown in Figures 6B–D, the calibration curve suggested that the predicted 1-, 3-, and 5-year overall survival was consistent with the actual 1-, 3-, and 5-year overall survival.

### Functional Analyses in the TCGA Osteosarcoma Cohort and GSE39055 Cohort

**FIGURE 7:**
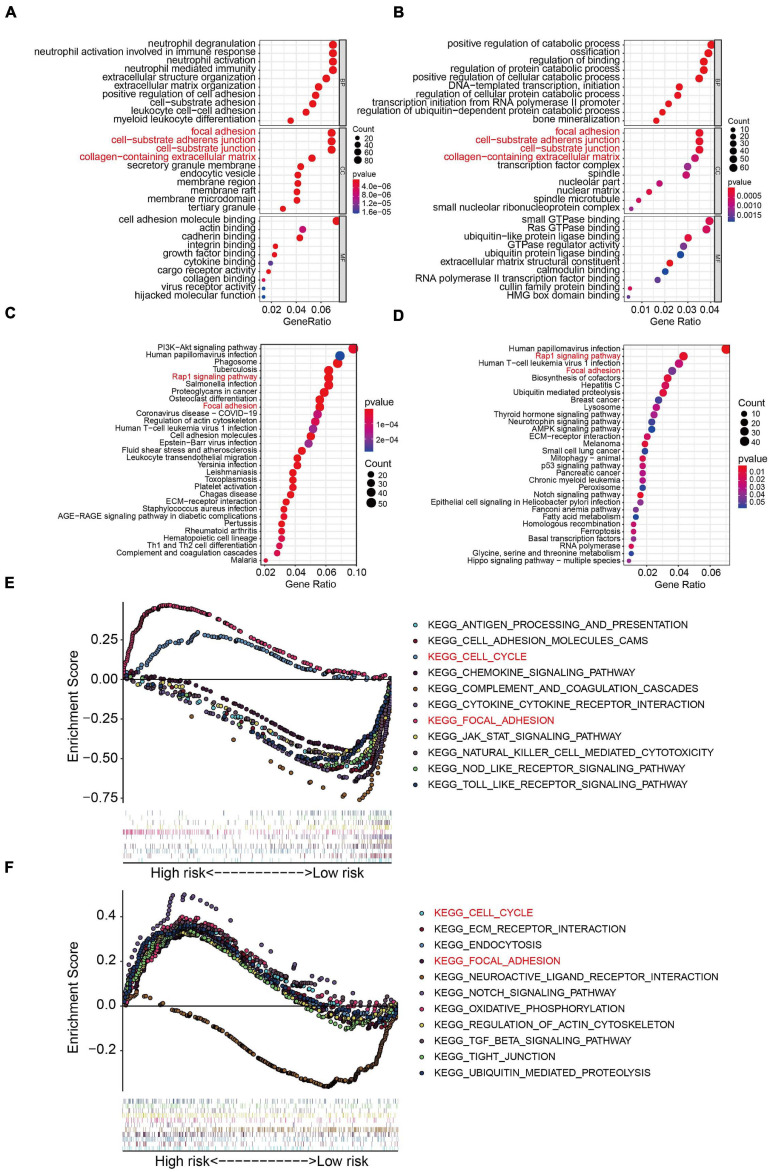
Functional analyses in the TCGA osteosarcoma dataset and GSE39055 dataset. **(A–B)** GO analysis of differentially expressed genes between the high- and low-risk groups in the TCGA osteosarcoma and GSE39055 datasets. **(C–D)** KEGG enrichment analysis of the differentially expressed genes in the TCGA osteosarcoma and GSE39055 datasets. **(E,F)** Gene set enrichment analysis in the TCGA osteosarcoma and GSE39055 datasets.

To further examine the biological processes and signaling pathways related to the risk score, we conducted functional enrichment analysis of genes differentially expressed between the high- and low-risk groups in the TCGA osteosarcoma and GSE39055 cohorts. GO enrichment analysis showed that cellular components including focal adhesion, cell-substrate adherens junction, cell-substrate junction, and collagen-containing extracellular matrix processes were significantly enriched in both the TCGA and GSE39055 cohorts (Figures 7A,B). Interestingly, KEGG pathway enrichment analysis also suggested that focal adhesion was one of the two pathways significantly enriched in the DEGs from the TCGA cohort and validated in the GSE39055 cohort, as was the Rap1 signaling pathway (Figures 7C,D). GSEA on the gene expression matrix of the TCGA cohort revealed that several cancer-related pathways including cell cycle and focal adhesion were significantly enriched in the high-risk group, and these enriched pathways were validated on the gene expression matrix of the GSE39055 cohort (Figures 7E,F).

### Differential Expression of the Six MRGs in Osteosarcoma

**FIGURE 8:**
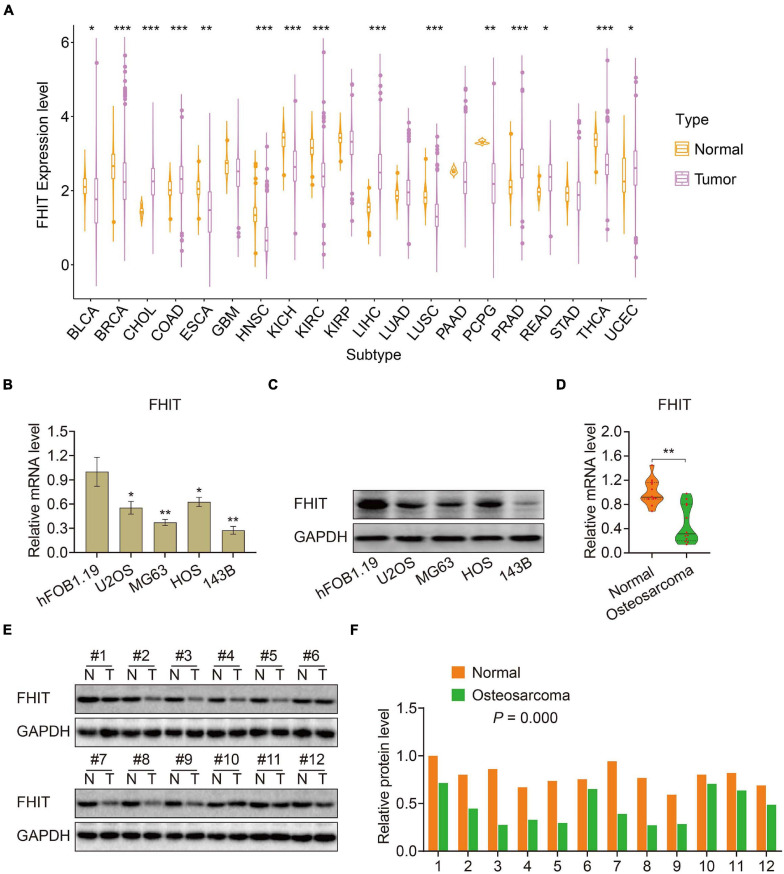
FHIT expression in osteosarcoma cells, tumor tissues, and other malignant tumors. **(A)** FHIT expression in other malignant tumors and adjacent normal tissues. **(B,C)** qRT-PCR and western blotting were performed to detect mRNA and protein expression of FHIT in osteosarcoma cells and normal osteoblasts. **(D,E)** mRNA and protein levels of FHIT were explored by qRT-PCR and western blotting. **(F)** Quantitative analysis of the western blot results. *P < 0.05, **P < 0.01, ***P < 0.001.

To elucidate the biological function of the six MRGs in osteosarcoma, we first analyzed their expression in primary and metastatic osteosarcoma samples in the GSE14359 and GSE32981 datasets. Expression of *ABCA3*, *CTGF*, and *AMIGO2* were significantly higher in metastatic osteosarcoma samples compared to the primary osteosarcoma samples (Supplementary Figures 1A–C) while the expression of *PREB*, *FHIT*, and *EXOSC5* were significantly decreased in metastatic osteosarcoma samples (Supplementary Figures 1D–F). Among the six MRGs, FHIT is a well-documented tumor suppressor and its expression was also downregulated in other malignant tumors including bladder, breast, esophageal cancer, head and neck squamous cell carcinoma, renal chromophobe cancers, renal clear cell carcinomas, lung squamous cell carcinomas, and thyroid carcinomas (Figure 8A). However, little is known about the role of FHIT in osteosarcoma. We therefore quantified the expression of FHIT in normal osteoblasts (hFOB1.19 cells) and osteosarcoma cells including 143B, U2OS, MG63, and HOS. qRT-PCR and western blotting showed that the mRNA and protein levels of FHIT were significantly lower in osteosarcoma cells compared to normal osteoblasts (Figures 8B,C). Consistently, FHIT mRNA and protein levels were significantly lower in osteosarcoma tissues than in adjacent normal tissues (Figures 8D–F).

### FHIT Suppresses Osteosarcoma Cell Proliferation and EMT

**FIGURE 9:**
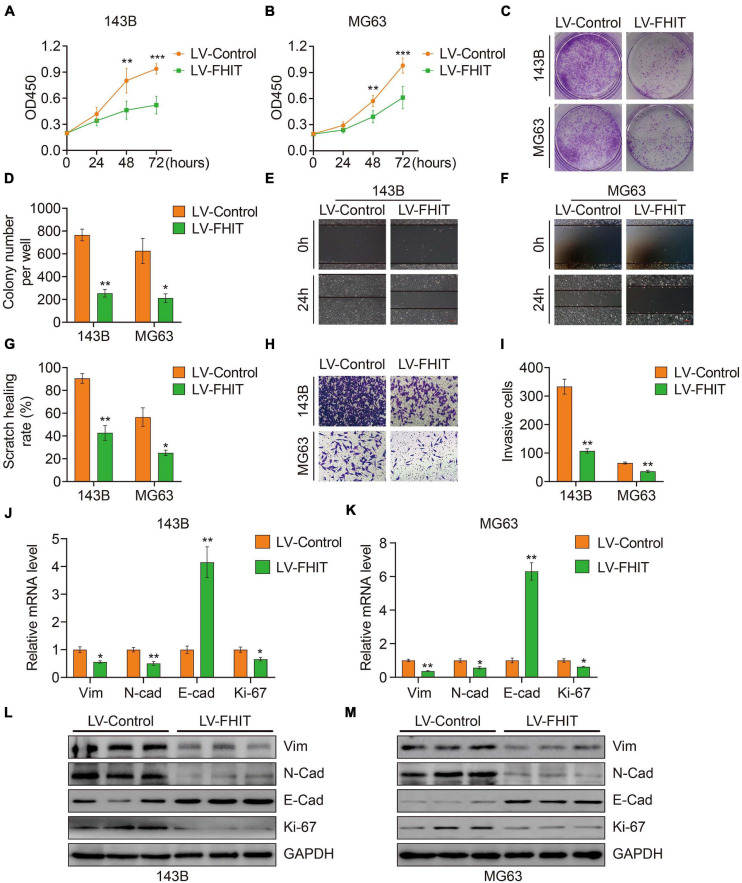
The effect of FHIT on osteosarcoma cell proliferation, migration, and invasion. CCK-8 **(A,B)** and colony formation assays **(C,D)** were used to assess the osteosarcoma cell proliferation. **(E–G)** A wound healing assay was performed to estimate the effect of FHIT overexpression on cell migration. Scale bar, 0.2 mm. **(H,I)** A transwell invasion assay was conducted to assess the effect of FHIT overexpression on osteosarcoma cell invasion. **(J,K)** The mRNA expression levels of EMT-related genes were detected by qRT-PCR. **(L,M)** Protein expression levels of vimentin, N-cadherin, E-cadherin, and Ki67 after overexpression of FHIT were measured by western blot analysis. *P < 0.05, **P < 0.01, ***P < 0.001.

To explore the role of FHIT in osteosarcoma, we first upregulated FHIT expression in 143B and MG63 cells by lentivirus transfection (Supplementary Figure 2) and then detected the effect of FHIT overexpression on cellular proliferation. As shown in Figures 9A–D, the CCK-8 and colony formation assays suggested that FHIT overexpression significantly inhibited 143B and MG63 cell proliferation. Cell migration and invasion are closely associated with osteosarcoma metastasis, so we further examined the effect of FHIT overexpression on osteosarcoma cell migration and invasion. As shown in Figures 9E–I, the migration and invasion of 143B and MG63 cells were significantly inhibited by FHIT overexpression. To further explore the mechanism underlying the inhibitory effect of FHIT in osteosarcoma, we quantified the expression of EMT related-genes in FHIT-overexpressing osteosarcoma cells. As shown in Figures 9J–M, FHIT overexpression decreased the mRNA and protein expression of Ki67, N-cadherin, and vimentin but increased expression of E-cadherin in osteosarcoma cells.

### FHIT Inhibits Tumor Growth *in vivo*

**FIGURE 10:**
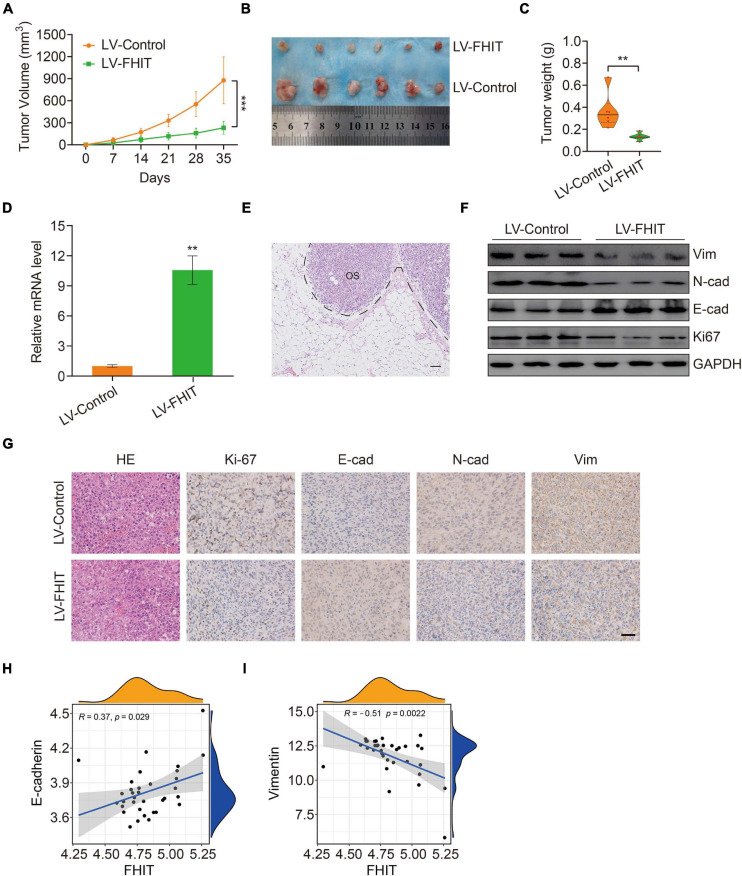
FHIT inhibits tumor growth *in vivo*. **(A)** Tumor volumes were monitored every week. **(B)** Subcutaneous tumors were dissected and photographed after 5 weeks. **(C)** Tumor weights are shown. **(D)** qRT-PCR was carried out to quantify the FHIT expression in subcutaneous tumor tissues. **(E)** Representative image of micro-metastasis in the lung tissues of nude mice injected with LV-Control 143B cells. OS, osteosarcoma. Scale bar, 1 cm. **(F)** Protein expression levels of vimentin, N-cadherin, E-cadherin, and Ki67 were measured by western blot analysis. **(G)** Immunohistochemistry analysis of Ki67, E-cadherin, N-cadherin, and vimentin in tumors. Scale bar, 0.5 cm. **(H,I)** The correlation of FHIT with E-cadherin and vimentin in GSE16091 dataset. **P < 0.01, ***P < 0.001.

Finally, we explored the effect of FHIT overexpression on osteosarcoma growth *in vivo*. FHIT-overexpressing 143B cells and control cells were injected subcutaneously into athymic nude mice and tumor volumes and weights were monitored. FHIT overexpression, confirmed by qPCR (Figure 10D), resulted in a significant reduction in tumor volume and weight (Figures 10A–C). Furthermore, micro-metastases were detected in the lung tissues of nude mice injected with LV-Control 143B cells (Figure 10E) but not in mice injected with LV-FHIT 143B cells. Furthermore, analysis of the expression of EMT-related genes in subcutaneous tumors by western blot and immunohistochemistry showed that Ki67, N-cadherin, and vimentin were significantly lower in the LV-FHIT group than in the LV-Control group, while E-cadherin expression markedly increased (Figures 10F,G). Moreover, analysis of the correlation of FHIT with EMT-related genes in GSE16091 dataset showed that FHIT was positively correlated with E-cadherin, while negatively correlated with vimentin (Figures 10H,I). These results suggest that FHIT inhibits epithelial-mesenchymal transition in osteosarcoma.

## Discussion

Metastasis is a typical hallmark of cancer and the main cause of osteosarcoma death (Hanahan and Weinberg, 2011; Moses et al., 2018). However, the mechanisms underlying osteosarcoma metastasis are largely unknown. Epithelial-mesenchymal transition (EMT) is a key process driving tumor metastasis (Mittal, 2018; Pastushenko and Blanpain, 2019). EMT is a dynamic and reversible process characterized by cells losing their epithelial-like features and transforming into cells with mesenchymal characteristics (Lu and Kang, 2019). Cells undergoing EMT have enhanced migratory capacity, contributing to their aggressive clinical behavior (Mittal, 2018). Though osteosarcoma cells are considered to arise from mesenchymal cells, recent studies have revealed that EMT occurs in osteosarcoma and contributes to its initiation, progression, and metastasis (Hou et al., 2014; Shen et al., 2017; Han et al., 2019; Xu et al., 2020). Many genes including EMT-related transcription factors and non-coding RNAs are associated with EMT and either promote or inhibit osteosarcoma metastasis. For example, the long non-coding (lnc)RNA *AFAP1-AS1* acts as an oncogene to promote EMT and osteosarcoma metastasis. Mechanistically, *AFAP1-AS1* could interact with RhoC, thereby activating the RhoC/ROCK1/p38MAPK/Twist1 signaling pathway (Shi et al., 2019). Plasma levels of miR-22 were also found to be an independent diagnostic and prognostic biomarker in osteosarcoma (Diao et al., 2020). Moreover, at the cellular level, miR-22 overexpression impaired proliferation and EMT in osteosarcoma by targeting Twist1 (Zhu et al., 2020). These studies suggest that EMT and distant metastasis are tightly regulated in osteosarcoma. However, the specific molecular events governing EMT and metastasis in osteosarcoma are still poorly understood.

Here we identified genes that were differentially expressed between primary and metastatic osteosarcoma, which we term MRGs. Functional analysis of the MRGs suggested that TNF and NF-kappa B signaling were significantly enriched in metastasis, two pathways that are known to regulate metastasis in a variety of tumors including osteosarcoma (Liao et al., 2015; Ren et al., 2017; Liu et al., 2020). We constructed a prognostic model for osteosarcoma patients comprising six MRGs that divided patients in both internal and external validation cohorts into high- and low-risk groups according to a median risk derived from patients in a training cohort. Kaplan-Meier survival analysis suggested that patients in the high-risk group had poor overall survival in the training cohort and all validation cohorts. To further evaluate the prognostic value of the model, Kaplan-Meier survival analysis was performed in various clinical sub-groups in the TCGA cohort. Consistently, in all clinical sub-groups, patients in the high-risk group had poorer overall survival than those in the low-risk group. ROC curve analysis confirmed the satisfactory accuracy of the prognostic model. Moreover, univariate and multivariate Cox regression analyses suggested that the risk score was independently prognostic in osteosarcoma patients. Taken together, these results suggest that the six-MRG signature had a robust prognostic value in osteosarcoma patients and was generalizable to other cohorts. A nomogram that included clinicopathological characteristics including age, gender, tumor site, and risk score was also designed to facilitate clinical decision-making.

To elucidate the molecular mechanism underlying osteosarcoma metastasis, we first identified those genes that were differentially expressed between high- and low-risk patients in the TCGA and GSE39055 cohorts. Functional enrichment analyses revealed that DEGs from both cohorts were significantly enriched in focal adhesion, suggesting a potential role in osteosarcoma metastasis. There is increasing evidence that focal adhesion is a vital determinant of cell migration and a promoter of tumor invasion and metastasis (Eke and Cordes, 2015; Shen et al., 2018). Thus, our analysis connected focal adhesion with osteosarcoma metastasis, a mechanism that requires further exploration.

The six MRGs used in the prognostic model were *ABCA3*, *CTGF*, *AMIGO2*, *PREB*, *EXOSC5*, and *FHIT*. The expression of the first three of these was increased in metastatic compared with primary osteosarcomas, while the latter three were decreased, suggesting differential functions for these genes in the metastatic process. ABCA3 is a highly conserved multi-membrane-spanning protein belonging to the ATP-binding cassette transporter superfamily and that plays a critical role in maintaining pulmonary surfactant homeostasis, with mutations confirmed to contribute to lung diseases including lung cancer (Beers and Mulugeta, 2017). Steinbach et al. (2006) established a link between aberrant ABCA3 expression and multi-drug resistance in childhood acute myeloid leukemia, suggesting a potential role for *ABCA3* in malignant tumors. However, until now, ABCA3 has not been implicated in tumor metastasis.

Connective tissue growth factor (CTGF) is a multifunctional signaling modulator that is increasingly recognized as promoting tumor metastasis by regulating cellular proliferation, migration, and invasion (Shen et al., 2020). Wang et al. (2017) recently reported that CTGF might promote osteosarcoma angiogenesis and metastasis by upregulating expression of Angpt2, consistent with our finding that CTGF is upregulated in metastatic osteosarcoma and is pro-metastatic. AMIGO2 (adhesion molecule with Ig-like domain 2) is a transmembrane protein with known oncoprotein function that is described as a driver of metastasis in breast and ovarian cancer and metastatic melanoma (Fontanals-Cirera et al., 2017; Sonzogni et al., 2018; Liu et al., 2021). The prolactin regulatory element binding (*PREB*) gene encodes a transcription factor that regulates target genes by binding to the prolactin gene (*PRL*) promoter (Zhang et al., 2017). PREB has described functions in hepatic glucose homeostasis (Park et al., 2018) and hepatitis C virus replication (Kong et al., 2016) but, to date, it is uncharacterized in cancer. Exosome component 5 (EXOSC5), also known as CML28 or Rrp46p, is an RNA exosome complex subunit overexpressed in various epithelial and hematopoietic tumor cell lines (Yang et al., 2002; Wu et al., 2005). In epithelial tumors such as colorectal cancer, EXOSC5 overexpression promotes proliferation by activating the ERK and AKT pathways (Pan et al., 2019), but its expression and role in mesenchymal tumors are unknown. Contrary to these previously described oncogenic functions, we found that EXOSC5 was downregulated in metastatic osteosarcoma so might have a tumor suppressor function in this tissue context.

The fragile histidine triad (*FHIT*) gene (located at 3p14.2) is a *bona fide* tumor suppressor. Hypermethylation of *FHIT* is commonly detected in human cancers, resulting in its inactivation as a very early event in cancer formation (Foja et al., 2005; Yan et al., 2016). Abnormal methylation or aberrant expression of *FHIT* has been shown to influence tumor cell proliferation, migration, invasion, and metastasis in a variety of cancers (Vecchione et al., 2004; Suh et al., 2014). FHIT expression is reduced in many breast cancers, and its nuclear translocation promotes breast cancer cell proliferation (Bianchi et al., 2015); moreover, loss of FHIT expression or function were correlated with patient poor prognosis and advanced stage (Campiglio et al., 1999; Arun et al., 2005). In lung cancer, *FHIT* hypermethylation, which inactivated *FHIT*, was significantly higher in tumors than in normal lung tissues. *FHIT* hypermethylation was also associated with an increased risk of lung cancer and a worse overall survival from the disease (Yan et al., 2016). Re-expression of FHIT in FHIT-negative highly invasive lung cancer cells inhibited migration and invasion, while FHIT knockdown in FHIT-positive poorly invasive cells increased their migratory or invasive potential (Joannes et al., 2010). This evidence indicates a critical role for FHIT in tumor metastasis. In osteosarcoma, Hinohara et al. (1998) reported abnormalities in *FHIT* transcripts but no functional analysis was performed. Xu et al. (2019) reported that FHIT expression was lower in osteosarcoma cells than that in normal osteoblasts and that its overexpression inhibited proliferation and promoted apoptosis of osteosarcoma cells. We found that FHIT overexpression reduced the migratory and invasive capacities of osteosarcoma cells. Mechanistically, FHIT overexpression repressed N-cadherin, vimentin, and Ki67 and increased the expression of E-cadherin, suggesting that its overexpression inhibits EMT in osteosarcoma cells. Our results suggest a metastasis-inhibiting potential for FHIT in osteosarcoma, making it a therapeutic target worthy of further investigation.

This study has some limitations. First, our MRGs were identified according to their differential expression between primary and metastatic osteosarcoma, which may have excluded some functional genes that were not differentially expressed in those tissues or at a different point in time. The detailed mechanism underlying how FHIT inhibits EMT also requires further exploration.

Nevertheless, we identified a novel six-MRG prognostic signature for osteosarcoma patients and a derived risk score that was a robust independent prognostic biomarker. Moreover, we constructed a nomogram to aid clinical decision-making and to personalize treatment for osteosarcoma patients. Of the six MRGs, FHIT is a potent tumor suppressor and its overexpression inhibited the migration, invasion, and metastasis of osteosarcoma by inhibiting EMT. FHIT therefore represents a promising therapeutic target in osteosarcoma patients.

## Data Availability Statement

Publicly available datasets were analyzed in this study. The names of the repository/repositories and accession number(s) can be found in the article/Supplementary Material.

## Ethics Statement

The studies involving human participants were reviewed and approved by the Ethics Committee of Wuhan University People’s Hospital. The patients/participants provided their written informed consent to participate in this study. The animal study was reviewed and approved by Ethics Committee of Wuhan University People’s Hospital.

## Author Contributions

WG and JY designed the study. KX, CG, and YQ completed the experiments. YS and WL analyzed the data. DZ conducted bioinformatic analysis, wrote the manuscript, and was responsible for language revisions. All authors reviewed the manuscript.

## Conflict of Interest

The authors declare that the research was conducted in the absence of any commercial or financial relationships that could be construed as a potential conflict of interest.

## Publisher’s Note

All claims expressed in this article are solely those of the authors and do not necessarily represent those of their affiliated organizations, or those of the publisher, the editors and the reviewers. Any product that may be evaluated in this article, or claim that may be made by its manufacturer, is not guaranteed or endorsed by the publisher.

## Footnotes

1 https://portal.gdc.cancer.gov/

2 https://www.ncbi.nlm.nih.gov/geo/

3 https://string-db.org/
