# Supplementary material for: A Novel Six Metastasis-Related Prognostic Gene Signature for Patients With Osteosarcoma
Source: Front Cell Dev Biol. 2021 Jul 23;9:699212. doi: 10.3389/fcell.2021.699212 (PMC8343004; doi:10.3389/fcell.2021.699212)
Supplement: Supplementary file 1 [file Data_Sheet_1.docx]

Supplementary Material


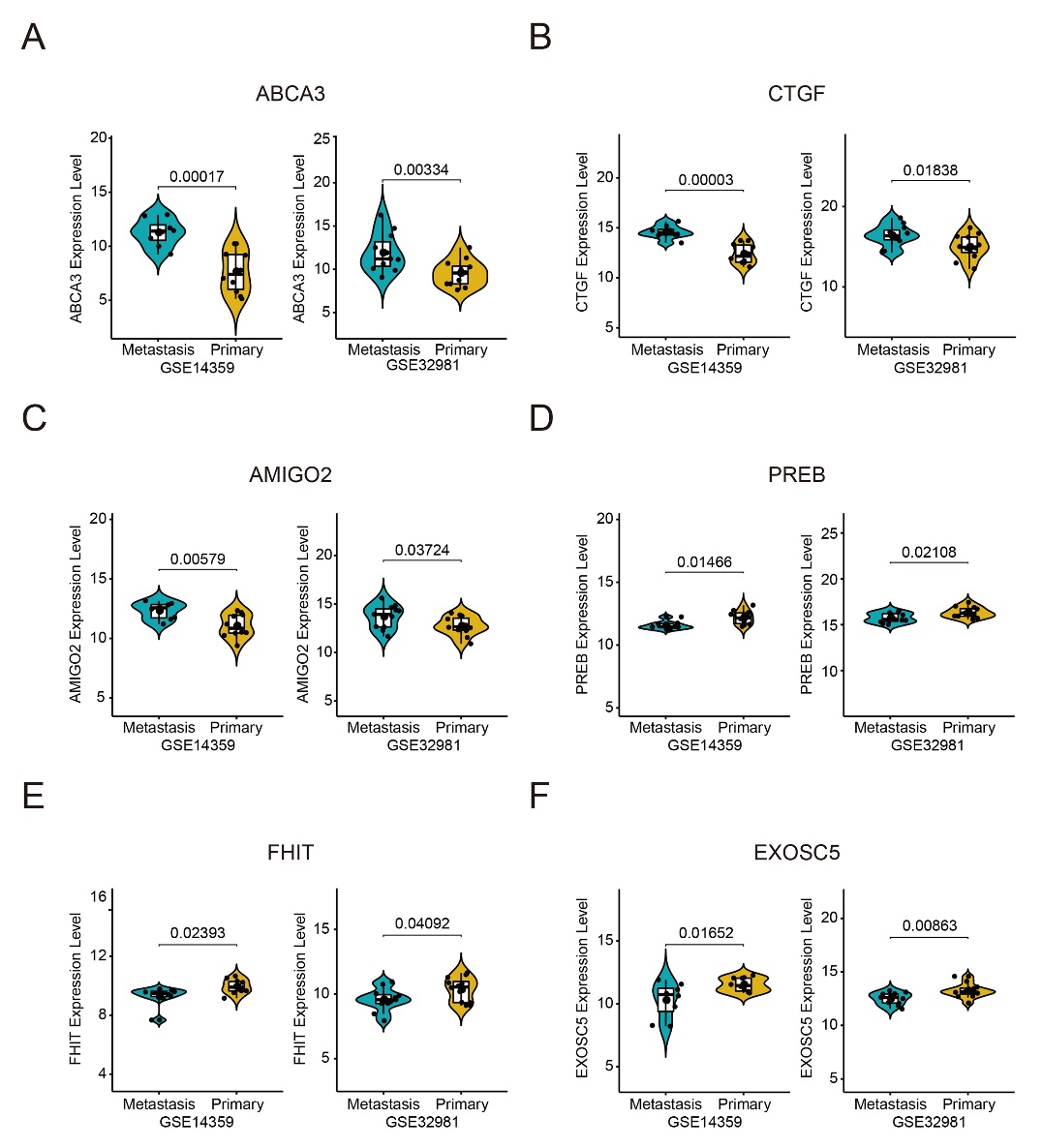


**Supplementary Figure 1.** The expression of the six MRGs in primary and metastatic osteosarcoma samples in the GSE14359 and GSE32981 datasets. (A-F) *ABCA3*, *CTGF*, *AMIGO2*, *PREB*, *FHIT*, and *EXOSC5* expression in primary and metastatic osteosarcoma.


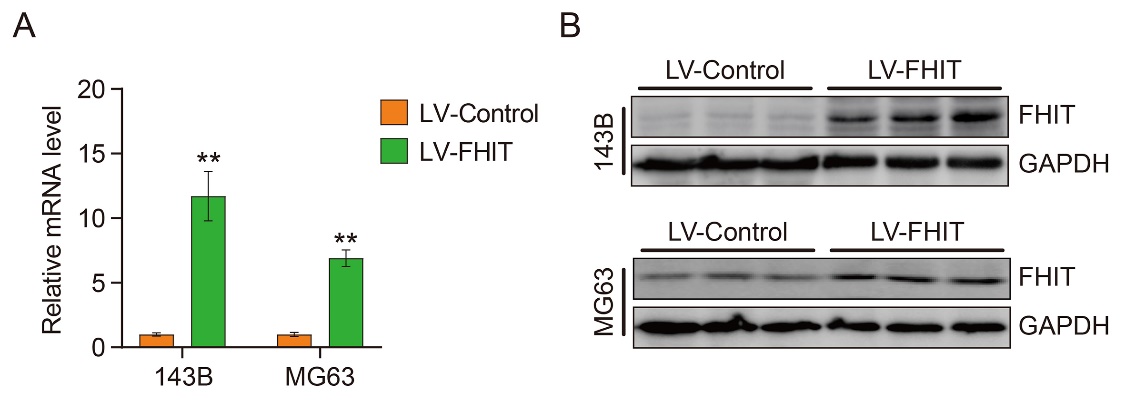


**Supplementary Figure 2.** The mRNA (A) and protein (B) expression levels of FHIT were significantly increased in stably transfected 143B and MG63 osteosarcoma cells.
